# Mcl-1 Inhibitor Induces Cells Death in BRAF-Mutant Amelanotic Melanoma Trough GSH Depletion, DNA Damage and Cell Cycle Changes

**DOI:** 10.1007/s12253-019-00715-z

**Published:** 2019-08-20

**Authors:** Michalina Respondek, Artur Beberok, Zuzanna Rzepka, Jakub Rok, Dorota Wrześniok

**Affiliations:** School of Pharmacy with the Division of Laboratory Medicine, Department of Pharmaceutical Chemistry, Medical University of Silesia, Jagiellońska 4, 41-200 Sosnowiec, Poland

**Keywords:** Amelanotic melanoma, Apoptosis, BH3 mimetics, MIM1, Dacarbazine, image cytometry

## Abstract

Mcl-1 is a potent antiapoptotic protein and amplifies frequently in many human cancer. Currently, it is considered that the extensively expressed of Mcl-1 protein in melanoma cells is associated with rapid tumor progression, poor prognosis and low chemosensitivity. Therefore, the antiapoptotic protein Mcl-1 could be considered as a potential target for malignant melanoma treatment. The aim of this study was to assess the effect of MIM1 a specific low molecular Mcl-1 protein inhibitor and mixture of MIM1 and dacarbazine on the viability, cell cycle progression and apoptosis induction in amelanotic C32 melanoma cells. The cytotoxic activity of MIM1 towards C32 melanoma cells was examined by the WST-1 test. The Mcl-1 protein level as a drug target in amelanotic melanoma cells was defined by Western blot analysis. Cell cycle progression, DNA fragmentation as well as GSH depletion were determined by fluorescence image cytometer NucleoCounter NC-3000. The obtained results demonstrate that the specific Mcl-1 protein inhibitor - MIM1 decreases cell viability and induce apoptosis (S-phase arrest, DNA fragmentation and redox imbalance) in amelanotic melanoma cells and intensify the proapoptotic properties of DTIC, as a result of interactions with Mcl-1 protein. Taken together, the presented data suggest that Mcl-1 protein is a an important target in malignant melanoma treatment and provide for the first time convincing evidence that MIM1, which inhibits Mcl-1 antiapoptotic protein is able to induce apoptosis and sensitize melanoma cells to alkylating agent.

## Introduction

Malignant melanoma, the most aggressive skin cancer occurs by malignant transformation of melanocytes. This type of cancer is characterized by rapid metastasis and resistance to standard therapy [1]. There are several types of melanoma and one of them is amelanotic melanoma with no pigmentation or with minimal residual pigmentation. The absence of pigment makes diagnosing very difficult and reduces patient’s chance to recover [2, 3]. Currently, standard treatment of malignant melanoma includes surgical resection, radiotherapy and systemic therapy such as immunotherapy, molecular therapy and chemotherapy with dacarbazine (DTIC) [4]. Despite the well-known of melanoma biology and advances in therapeutic approaches, the 5-years survival of patients at an advanced stages is only 14%. Therefore effective treatment of metastatic melanoma is still remains a challenge [5].

Apoptosis dysfunction is a hallmark of cancer and constitutes an important mechanism in tumor development, progression and therapeutic resistance [6]. This led to the development of new anticancer strategies targeting apoptosis such as the inhibition of prosurvival proteins that are overexpressed in malignancies [7, 8]. Currently, there are known specific small molecules called BH3 mimetics which mimics BH3-only proteins and inhibits antiapoptotic proteins Bcl-2 thus inducing apoptosis. The effectiveness of BH3 mimetics in apoptosis induction depend on the selection of adequate BH3 mimetic able to suppress Bcl-2 protein which plays a key role in survival of specific type of cancer [9, 10].

Malignant melanoma cells exhibit high level and activity of antiapoptotic Mcl-1 protein [11, 12]. Currently data indicate that elevated expression of this protein is associated with rapid melanoma progression as well as resistance to chemotherapeutic agents [13, 14]. Moreover, recent studies indicate that reduction of Mcl-1 protein in melanoma cells are necessary and sufficient to induce programmed cell death [15, 16]. Therefore, Mcl-1 protein could constitute important with high priority target for melanoma treatment including advanced stages.

MIM1 (4-((E)-((Z)-2-(cyclohexylimino)-4-methylthiazol-3(2H)-ylimino)methyl) benzene 1,2,3-triol) is high specific Mcl-1 inhibitor which presents favourable biophysical as well as biological properties e.g. low molecular weight, desired solubility and stability. Moreover, it was found that MIM1 can selectively inhibit Mcl-1 protein and induce caspase 3/7 activation and cell death in Mcl-1-dependent leukemia cells [17, 18].

Presently, there are no information about the proapoptic properties of this small molecule towards amelanotic melanoma cells. Therefore, in the present study we investigated, for the first time, the impact of MIM1, alone or in combination with DTIC, on the viability, apoptosis and cell cycle progression in C32 amelanotic melanoma cells.

## Materials and Methods

### Materials

MIM1 (4-((E)-((Z)-2-(cyclohexylimino)-4-methylthiazol-3(2H)-ylimino)methyl) benzene 1,2,3-triol), was purchased from Merck Millipore (Germany). Dacarbazine (DTIC), dimethyl sulphoxide (DMSO), amphotericin B and penicillin were obtained from Sigma-Aldrich Inc. (USA). Dacarbazine (DTIC) was activated by exposure to light for 1 h. Growth medium DMEM, fetal bovine serum as well as trypsin/EDTA were obtained from Cytogen (Poland). WST-1 reagent was purchased from Roche GmbH (Germany). Solution 3 (1 μg/ml DAPI, 0.1% triton X-100 in PBS), Solution 5 (VB-48TM, propidium iodide—PI, acridine orange—AO), were obtained from ChemoMetec (Denmark). Immunoblot analysis was performed using: Mcl-1 Antibody (4572), GAPDH Antibody (14C10) from Cell Signaling (Danvers, MA, USA), Anti-Rabbit IgG (A154), Tween-20, RIPA Buffer and PVDF membranes from Sigma-Aldrich Inc. (USA). BCA Protein Assay Kit and ECL Western Blotting Substrate were purchased from Thermo Fisher Scientific (USA). The remaining chemicals were produced by POCH S.A. (Poland).

### Cell Culture

The human amelanotic melanoma cells C32 reveals a BRAF mutation signature (V600E) (American Type Culture Collection – ATCC, CRL-1974, Manassas, VA, USA) were cultured in high-glucose DMEM supplemented with 10% heat-inactivated fetal bovine serum, penicillin (10.000 U/ml), neomycin (10 μg/ml) and amphotericin B (0.25 mg/ml) at 37 °C in 5% CO_2_.

### Cell Viability Assay

The cell viability was evaluated using the WST-1 (4-[3-(4- iodophenyl)-2-(4-nitrophenyl)-2H-5-tetrazolio]-1,3-benzene disulphonate) colorimetric assay. Human amelanotic melanoma cells were plated at 2500 cells per well in 96-well microplate and preincubated in DMEM for 24 h at 37 °C and 5% CO_2_. Then the medium was removed and cells were treated with MIM1 or DTIC in concentration range 1.0–50 μM and MIM1/DTIC mixture (1:1, 50 μM). The WST-1 reagent was added into each well 3 h before measurement. The absorbance was measured at 440 nm using a microplate reader Infinite 200 PRO (TECAN, Switzerland). A reference wavelength was used at 650 nm. The controls were normalized to 100% for each assay and treatments were expressed as the percentage of the controls.

### Vitality Assay – Analysis of the Level of Cellular Reduced Glutathione (GSH)

The intracellular GSH level in C32 amelanotic melanoma cells was measured using fluorescence image cytometer NucleoCounter NC-3000. Cells were incubated with MIM1, DTIC or MIM1/DTIC mixture (1:1) in concentration 50 μM for 24 h, 48 h and 72 h. Then the studied cells were harvested by trypsinization, centrifuged and quantified. Solution 5 containing VB-48TM, propidium iodide and acridine orange was added into the each samples. The stained cells were analyzed using NucleoView NC-3000 software (version 1.4, ChemoMetec).

### DNA Fragmentation Assay

DNA fragmentation, a key event of late apoptosis, was detected by the use of the fluorescence image cytometer NucleoCounter NC-3000. C32 cells were treated with MIM1, DTIC or MIM1/DTIC mixture (1:1) in concentration 50 μM. After 24 h, 48 h or 72 h of incubation with studied agents, the cells were harvested using trypsin/EDTA, quantified and fixed with 70% cold ethanol for at least 12 h. Then the ethanol was removed, cells were washed with PBS and stained with 1 μg/ml DAPI (Solution 3 containing DAPI and Triton X-100 to trigger cellular membrane damage). The stained cells were analyzed using NucleoView NC-3000 software (version 1.4, ChemoMetec). Cellular fluorescence was quantified and apoptotic cells with fragmented DNA were seen as a sub-G1 peak in a DNA content histogram.

### Fixed Cell Cycle-DAPI Assay

Cell cycle analysis of C32 cells was performed by the use of the fluorescence image cytometer NucleoCounter NC-3000 (Denmark). The amelanotic melanoma cells were cultured with MIM1, DTIC or MIM1/DTIC (1:1) mixture in concentration 50 μM for 24 h, 48 h and 72 h. After incubation, the cells were tryspinized, quantified and fixed with 70% cold ethanol at 4 °C for 24 h. Then the ethanol was removed and the cells were washed with PBS. To the cell pellets Solution 3 (containing DAPI and Triton X-100 to trigger cellular membrane damage) was added and the samples were incubated for 5 min of at 37 °C. The stained cells were analyzed using the NucleoView NC-3000 software, version 1.4. Obtained results were presented in the DNA content histograms where different phases of the cell cycle were demarcated.

### Cell Morphology Assessment

To investigate the morphological changes induced by MIM1, DTIC and MIM1/DTIC mixture, C32 cells were seeded in T-25 flasks (3 × 10^6^ cells/flask) in DMEM supplemented medium. Incubation with MIM1, DTIC or MIM1/DTIC mixture (1:1) in concentration 50 μM began 24 h after seeding. The cells were observed after 24 h, 48 h and 72 h of treatment with studied agents, under the light inverted microscope NIKON TS100F (Japan) and morphological changes were recorded.

### Western Blot Analysis

Cells were lysed in RIPA buffer with protease and phosphatase inhibitors. The lysates were incubated on ice for 30 min. After that the protein concentration was quantitated spectrophotometrically (Denovix DS-11) using BCA protein assay. Protein lysates (45 μg/lane) were separated on 10%. SDS-PAGE and than transferred onto polyvinylidene fluoride membrane. The transferred membrane was blocked in TBS-T (0.1% TBS supplemented with Tween-20) with 5% dry milk by 1 h, washed and incubated with primary antibodies overnight at 4 °C. After incubation with primary antibody, the membrane was rinsed well and incubated with horseradish peroxidase-conjugated secondary antibody by 1.5 h at room temperature. The protein signals were detected using ECL chemiluminescence reagent. Immunoreactive proteins were visualized using via G:Box Chemi-XT4 Imaging System. Densitometry measurements were made using GeneTools Software (version 4.3.5), normalized to GAPDH content and finally presented as the percentages of the control.

### Statistical Analysis

In all experiments, mean values of at least three separate experiments performed in triplicate (*n* = 9) ± standard error of the mean (SEM) were calculated. The results were analyzed statistically using GraphPad Prism 6.01 Software by means of one-way ANOVA as well as Dunnett’s comparison test. In all cases the statistical significance was found for *p* value lower than 0.05.

## Results

### The Effects of MIM1, DTIC or MIM1/DTIC Mixture on Cell Viability

**Fig. 1:**
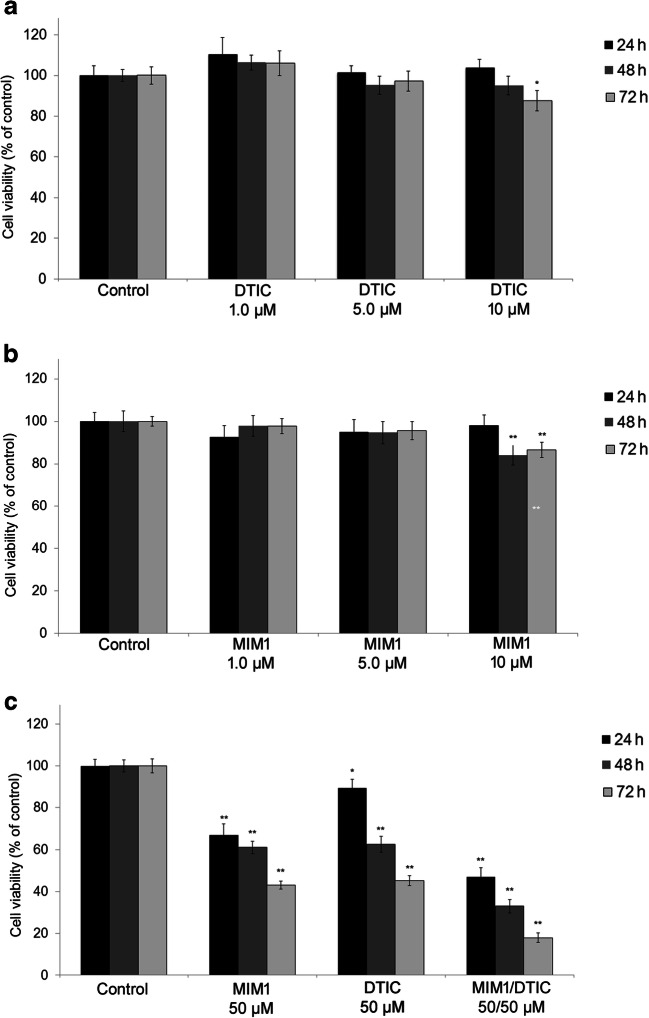
The effect of MIM1, DTIC and MIM1/DTIC mixture on the viability of C32 cells. **a** The cells were treated with increasing concentrations of DTIC (1.0 μM – 10 μM) for 24 h, 48 h and 72 h. Data are presented as % of the controls. *p < 0.05. **b** The cells were treated with increasing concentrations of MIM1 (1.0 μM – 10 μM) for 24 h, 48 h and 72 h. Data are presented as % of the controls. *p < 0.05, ** *p* < 0.01. **c** The cells were treated with MIM1, DTIC and MIM1/DTIC mixture (1:1) in concentrations 50 μM for 24 h, 48 h and 72 h. Data are presented as % of the controls. The cell viability was determined using WST-1 assay. *p < 0.05, ** *p* < 0.01

The WST-1 (4-[3-(4-iodophenyl)-2-(4-nitrophenyl)-2H-5-tetrazolio]-1,3-benzenedisulphonate) colorimetric test was performed to assess the effect of MIM1, DTIC or MIM1/DTIC mixture on C32 cell viability. There was a high decrease of cell viability after single and combined treatments with MIM1 and DTIC, as compared to control (Fig. 1c). Treatment of C32 cells with DTIC in concentration 50 μM for 24 h, 48 h and 72 h decreased cell viability by 11%, 38% and 55%, respectively. Following incubation of cells with MIM1 in concentration 50 μM for 24 h, 48 h and 72 h the loss in cell viability was about 31%, 39% and 57%, respectively. In the lower drug concentrations of MIM1 or DTIC (from 1.0 μM to 5 μM) the loss in cell viability was not statistical significant whereas the use of these agents in concentration 10 μM for 48 h and 72 h resulted in decrease of cell viability by about 15–17%, as compared with the controls (Fig. 1a and b). The highest cytotoxic effect toward C32 cells was observed after combined treatment with the studied agents (1:1, 50 μM) with the reduction in the cells viability by 53%, 67% and 82% for 24 h, 48 h and 72 h incubation time, respectively.

### The Influence of MIM1, DTIC and MIM1/DTIC Mixture on Cellular GSH Level

**Fig. 2:**
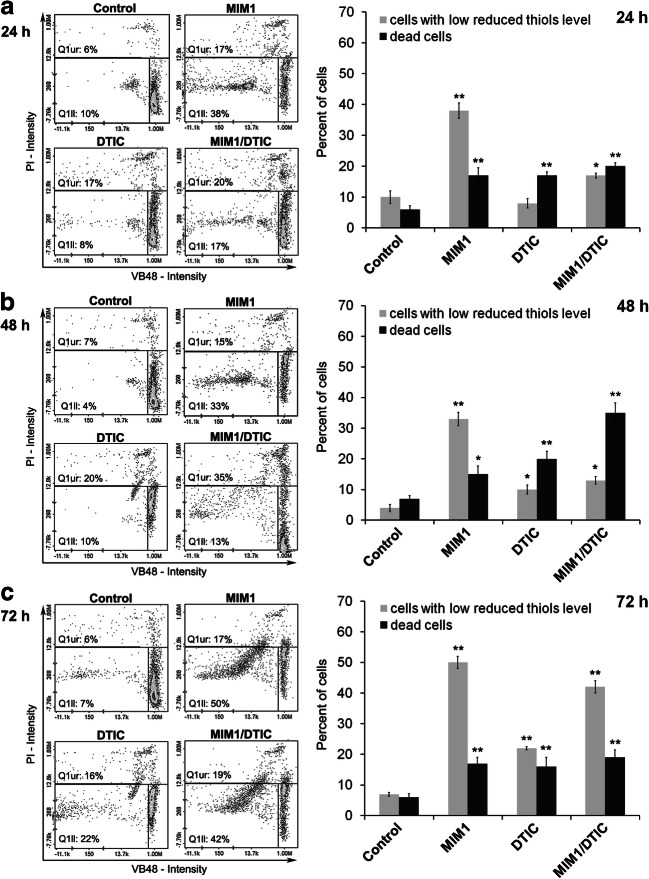
Cellular GSH level in C32 cells after the exposure to MIM1, DTIC or MIM1/DTIC mixture for (**a**) 24 h, (**b**) 48 h and (**c**) 72 h. The presented histograms are representative for three independent experiments with similar results. Q1ur — PI-positive cells (dead cells); Q1ll — cells with low gluthathione level. Bar graph showing the percentages of cells with the cellular reduced glutathione level after incubation with MIM1, DTIC and MIM1/DTIC mixture for 24 h, 48 h and 72 h. Bar graph represents mean ± SEM from three independent experiments. **p < 0.01

A cellular GSH depletion correlates well with an apoptosis progression. Quantification of the intracellular GSH level in the amelanotic melanoma cells after incubation with MIM1, DTIC or MIM1/DTIC mixture was determined using fluorescence image cytometry (Fig. 2). The exposure of C32 cells to DTIC slightly increased the percentages of cells with low reduced the thiols level (by 15% after 72 h of incubation) and dead cells (by 10%, 14% and 6% after 24 h, 48 h and 72 h of incubation, respectively). The treatment with MIM1 for 24 h, 48 h and 72 h greatly increased the percentages of cells with low vitality by 30%, 32% and 50%, respectively. Simultaneously, there was only weak increase in the percentage of dead cells (about 15%). After treatment of C32 cells with the mixture for 24 h, 48 h and 72 h the percentages of cells with low vitality increased by 7%, 9% and 35%, respectively as compared with the controls. At the same exposure conditions a significant increase in the percentages of dead cells was noticed (by 14%, 28% and 13% after 24 h, 48 h and 72 h of incubation time, respectively).

### MIM1 and MIM1/DTIC Mixture Induce DNA Fragmentation

**Fig. 3:**
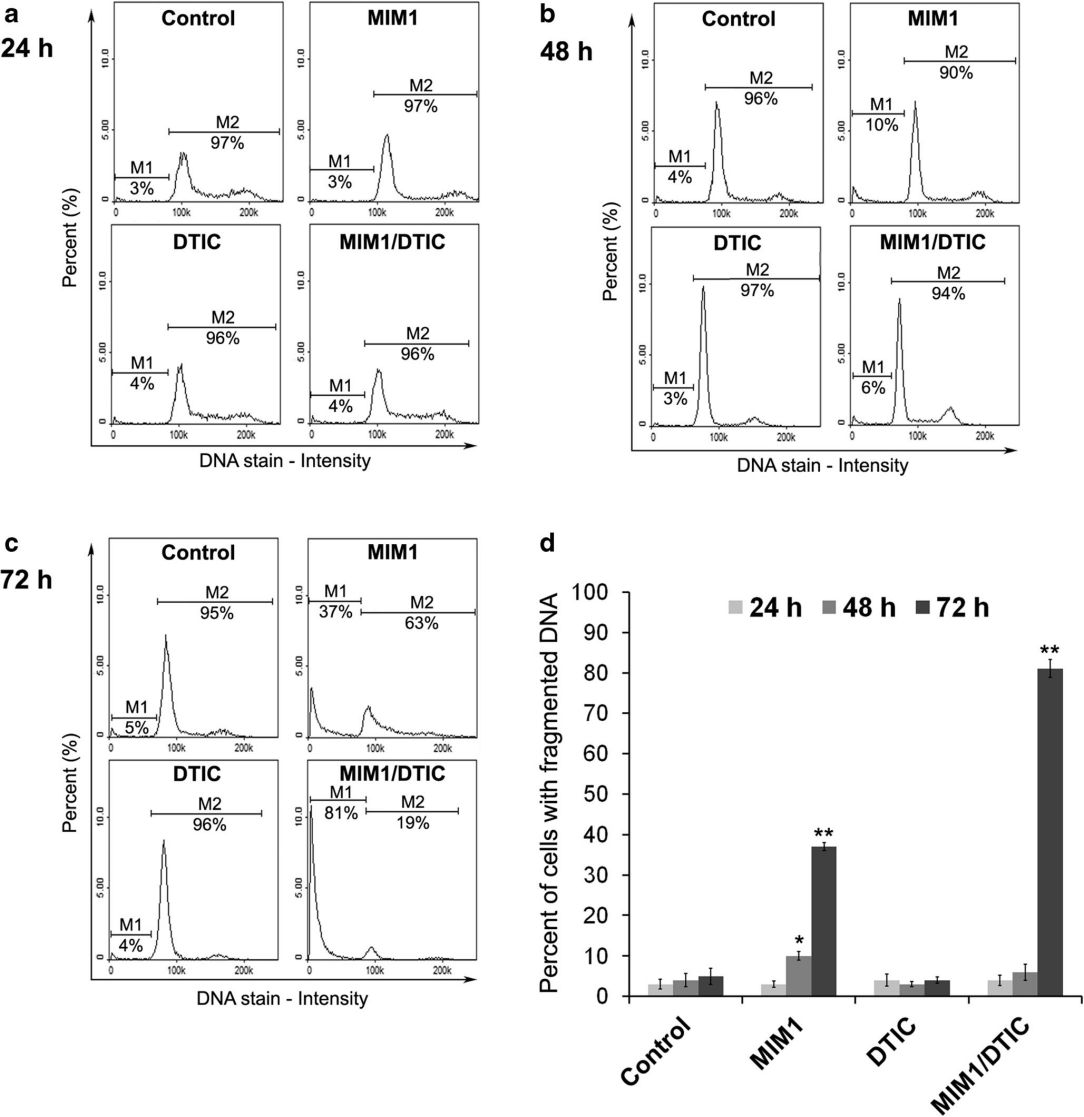
MIM1 and MIM1/DTIC mixture caused DNA fragmentation in C32 cells. The cells were treated with MIM1, DTIC or the mixture for (**a**) 24 h, (**b**) 48 h and (**c**) 72 h. Data are presented as the percentages of cells with fragmented DNA. Presented histograms are representative for three independent experiments with similar results. M1— the cells with fragmented DNA (having less than one DNA equivalent); M2— the cells having one or more than one DNA equivalent. **d** A graph shows the percentages of cells having less than 1 DNA equivalent after incubation with MIM1, DTIC or the mixture for 24 h, 48 h and 72 h. The bar graph represents mean ± SEM from three independent experiments. **p < 0.01

Cleavage of chromosomal DNA is an integral part of apoptosis and a key apoptotic marker. The effect of MIM1, DTIC or MIM1/DTIC mixture on the DNA fragmentation was performed with the use of fluorescence image cytometer and DNA content assay (Fig. 3). The DNA fragmentation in C32 cells was detected after 48 h and 72 h of incubation with MIM1: the percentages of cells with fragmented DNA found to be as 10% and 37%, respectively, while the values determined for the controls were 3% and 4%, respectively. The highest induction of DNA fragmentation in C32 cells was observed after 72 h of incubation with the mixture where percentage of cells with fragmented DNA was 81%. In contrast, after exposure of the cells to DTIC alone the DNA fragmentation was not observed.

### MIM1 and MIM1/DTIC Mixture Cause Changes in Cell Cycle Progression

**Fig. 4:**
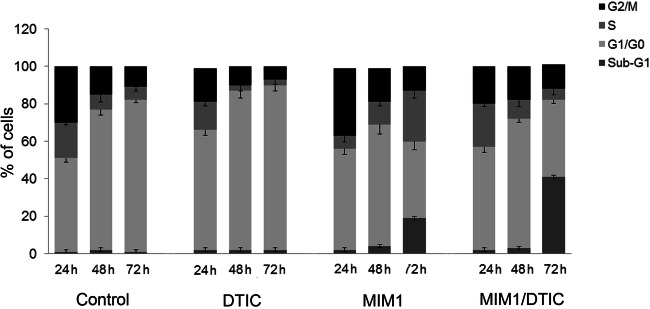
MIM1, DTIC or MIM1/DTIC mixture induced changes in cell cycle progression. The cell cycle analysis of C32 cells treated with the studied agents was performed after 24 h 48 h and 72 h of incubation. The results are presented as the percentages of the cells in different cell-cycle phases. The bar graph represents mean ± SEM from three independent experiments

Cell cycle profile of C32 cells was determined after exposure to MIM1, DTIC or MIM1/DTIC mixture. The cell cycle analysis was performed using fluorescence image cytometer (Fig. 4). Treatment C32 cells with MIM1 for 72 h caused arrest in S- and Sub-G1 phases. The percentages of S- and Sub-G1 fraction increased from 7% to 27% and from 1% to 19%, respectively as compared with the controls. Simultaneously, DTIC treatment slightly increased the number of cells in G1/G0 phase. The percentages of G1/G0 fraction after 24 h and 48 h treatment with DTIC were 64% and 85%, respectively, while the values of the controls were 50% and 75%. Changes in cell cycle progression after MIM1/DTIC mixture treatment were also observed and after prolongation of incubation time up to 72 h, the cells demonstrated significant increase in sub-G1 peak (apoptotic cells) from 2% to 41%.

### Morphological Changes

**Fig. 5:**
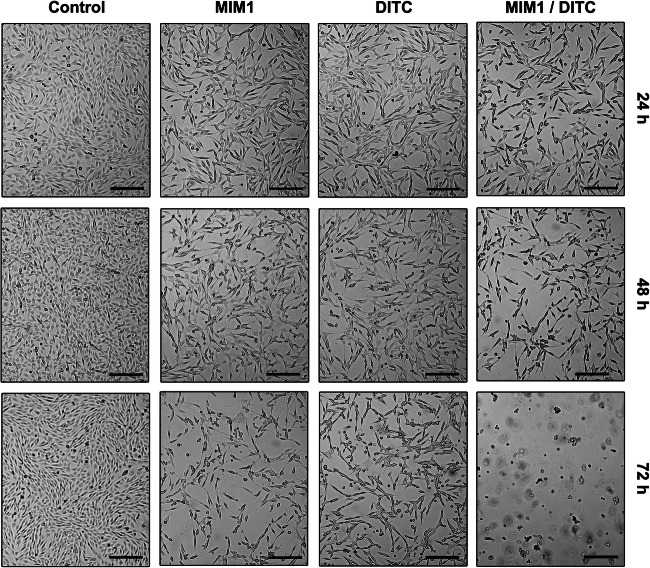
MIM1 and MIM1/DTIC mixture causes morphological changes in C32 cells. The cells were treated with MIM1, DTIC or MIM1/DTIC mixture for 24 h, 48 h and 72 h treatment. The amelanotic melanoma cells were observed under the light inverted microscope (scale bar = 40 μm)

The morphological changes of the amelanotic melanoma cells were recorded using light inverted microscope at 40× magnification. As shown in Fig. 5, the untreated cells present original properties such as adherent grew with normal size and cell-cell cohesion. C32 cells treated with MIM1, DTIC and MIM1/DTIC mixture for 24 h, 48 h and 72 h expressed characteristic features, like increase in cell volume, loss of cell-cell connection. Moreover, a decrease in cell number was observed. After prolongation of incubation time up to 72 h with MIM1 or MIM1/DTIC mixture, the cells also displayed features like rounding, loss of the cell volume, shrinkage and membrane blebbing which are known as the apoptosis symptoms.

### Mcl-1 Protein Level

**Fig. 6:**
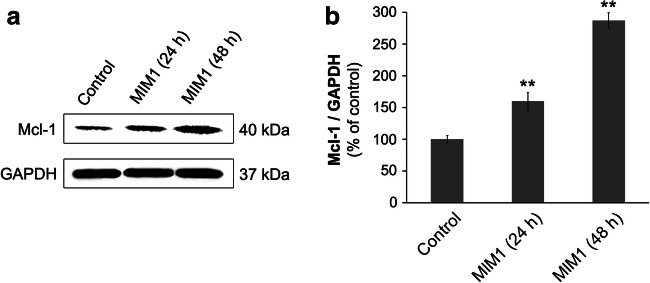
**a** Western blot analysis of basal Mc-1 level in C32 cells and Mcl-1 levels in the cells incubated with 50 μM MIM1 for 24 h and 48 h. GAPDH was used as a cell protein control. Blots are representative of three independent experiments. **b** Western blot analysis of Mcl-1 in C32 cells treated with 50 μM MIM1 for 24 h and 48 h. Data are presented as % of the control. ** *p* < 0.01

Mcl-1 protein level as drug target in amelanotic melanoma cells was determined by Western blotting. The loss of cell viability and apoptosis induction in C32 melanoma cells after selective Mcl-1 inhibitor treatment, may be associated with overexpression of Mcl-1 protein in studied cells. In agreement with this hypothesis, we detected Mcl-1 protein in C32 amelanotic melanoma cells (Fig. 6). Moreover, treatment with MIM1 in concentration 50 μM increased the level of Mcl-1 protein in studied cells in a time dependent manner by 60% for 24 h and by 180% for 48 h.

## Discussion

Development and progression of malignant melanoma mainly result from dysfunction of pro- and antiapoptotic Bcl-2 family proteins, especially antiapoptotic Mcl-1 protein [13, 14]. There are several agents that inhibit activity of antiapoptotic proteins by imitating endogenous proapoptotic BH3-only proteins. Many of these agents have been tested in in vitro and in vivo studies and as well as in clinical trials [7, 9]. However only few of them have been examined on melanoma cells. MIM1 is a potent and selective small molecule capable to block Mcl-1 protein activity [17].

To our knowledge, the cytotoxic effect of MIM1 towards an amelanotic melanoma cells has not been reported in literature. The present study is the first that shows the capacity of specific Mcl-1 protein inhibitor – MIM1 to induce apoptosis in the amelanotic melanoma cells with BRAF ^V600E^ mutation and to intensify the proapoptotic properties of DTIC. The obtained results shown that amelanotic melanoma cells are susceptible to specific Mcl-1 inhibitor. MIM1 as well as DTIC decreased the cell viability in a dose- and time-dependent manner. Following treatment of C32 cells with MIM1 in concentration 50 μM for 72 h, the cell viability decreased by 42% (Fig. 1c). About 55% decrease in the viability of studied cells was noticed for DTIC at the same exposure conditions (Fig. 1c), what indicated that MIM1 shows similar cytotoxic effect towards amelanotic melanoma cells as the reference drug. The obtained results as well as accessible literature data indicate that antiapoptotic Mcl-1 protein seems to be crucial in melanoma cells death and survive [15, 16, 19]. Moreover, high level of Mcl-1 protein may be associated with melanoma resistance to standard chemotherapeutic agents [13, 20] Thallingher et al. [20] in in vivo study demonstrated that simultaneous dawnregulation of Mcl-1 protein and dacarbazine treatment resulted in enhanced tumor cell apoptosis and significant reduced mean tumor weight. Interestingly, the present study also confirms the hypothesis that Mcl-1 may be one of the key factor that influences the chemosensitivity of malignant melanoma. The results of our study revealed that the combination of MIM1 and DTIC strongly reduced cell viability as early as 24 h exposure (by 53%) and this effect was intensified after prolongation of incubation time up to 72 h where the cell viability decreased by 82%. Thus it may pointed out that the potent cytotoxic effect of the tested mixture occurs earlier than other single treatment of cells with MIM1 or DTIC alone (Fig. 1c).

To evaluate whether the demonstrated decrease in C32 cells viability induced by the studied agents may be due to apoptosis induction, the intracellular GSH level and DNA fragmentation were examined. Intracellular GSH depletion enhances the sensitivity of cancer cells to apoptosis induced by chemical agents and ionizing radiations [21]. Moreover, mitochondrial GSH oxidation favours mitochondrial membrane permeability, thus facilitating the release of proapoptotic factors and directly induce or potentiate apoptosis [22]. In the present study, the depletion of intracellular GSH in the amelanotic melanoma cells was noted following exposure to MIM1, DTIC as well as MIM1/DTIC mixture (Fig. 2). Thus it demonstrated that MIM1, MIM1/DITC mixture as well as DTIC may induce apoptosis in C32 cells through alteration of redox signalling pathways. Moreover, the results of intracellular GSH loss may be a consequence of an oxidative stress [23]. Directly connection of the oxidative stress induction and GSH depletion in melanoma cells has been shown by Beberok et al. [24], where reactive oxygen species (ROS) generation correlated well with GSH depletion in melanoma cells after lomefloxacin exposure. It indicates that the observed GSH depletion in the melanoma cells after MIM1 or MIM1/DTIC mixture treatment may be also associated with the overproduction of ROS.

To detect the final execution phase of apoptosis, the DNA fragmentation in the amelanotic melanoma cells was examined. The obtained results confirm that C32 cells after exposure to MIM1 or MIM1/DTIC mixture enter into the final phase of programmed cell death (Fig. 3). A more marked increase in the percentages of cells with fragmented DNA after the tested mixture treatment (81% of cell with fragmented DNA) as compared with the use MIM1 alone (37% of cells with fragmented DNA). Pointed out that simultaneously exposure of C32 cells to MIM1 or DTIC intensified the proapoptotic effect. Interestingly, the use of DTIC in the tested exposure conditions did not cause DNA fragmentation in C32 cells.

The cell cycle governs cell proliferation and plays a key role in the oncogenesis and apoptosis [25]. Zhao et al. [15] showed that another Mcl-1 inhibitor-demethylzeylasteral inhibited Mcl-1 protein and accelerated the ubiquitin-dependent degradation of CDK2 in melanoma cells which resulted in cell cycle arrest at S phase. In the present study, the impact of MIM1, DTIC and MIM1/DTIC mixture on cell cycle progression in C32 cells was examined. The obtained results demonstrated that after prolongation of incubation time up to 72 h, MIM1 caused S- phase arrest in the studied cells (Fig. 4). The MIM1 caused also accumulation of C32 cells in Sub-G1 phase. The Sub-G1 fraction represents cells in late apoptotic phase with fragmented DNA [26]. These findings are consistent with results obtained from DNA fragmentation assay (Fig. 3). In opposite to MIM1, the DTIC treatment caused significantly increased in the percentage of C32 cells in G1/G0 phase. Similarly, Al-Qatati and Aliwaini [27] presented that melanoma cells treatment with DTIC primarily led to cell cycle arrest in the G1 phase. Our data also demonstrated that MIM1/DTIC mixture causes sub-G1 phase arrest in C32 cells. The Sub-G1 fraction which represents cells in late apoptotic phase [26] is the highest after mixture exposure. These findings are consistent with results obtained from the viability test as well as DNA fragmentation assay.

Given the experimental evidence from our study, MIM1 – a selective Mcl-1 protein inhibitor, is a potent apoptosis inducer in the amelanotic melanoma cells. This observation indicates that Mcl-1 protein could be considered as a key factor that influences on the chemosensitivity and programmed cell death. The main role of Mcl-1 protein was also confirmed by the data showing the sensitivity of this cells to other BH3 mimetics.

To determine whether the loss of the cell viability and apoptosis induction in amelanotic melanoma cells may be associated with the interaction of MIM1 with Mcl-1 protein, the level of Mcl-1 in C32 cells was determined. Based on the results of western blot analysis the presence of Mcl-1 protein has been distinctly demonstrated in studied cells. Moreover, the exposure of C32 cells to MIM1 treatment caused significant, which intensified after prolongation of incubation time. Similarly, Luedtke et al. [28] and Caenepeel et al. [29] presented that treatment of many cancer cell lines (A427, MV-4-11, and NCI-H1568, U266B1, THP-1 and U937) with Mcl-1 inhibitors - A-1210477 or AMG 176 lead to increase of Mcl-1 protein level. This phenomenon was associated with the release of Bim from the formed complexes with Mcl-1 protein. Bim is a BH3-only proapoptotic protein leading to Bax/Bak activation and finally causing apoptosis. Therefore, presented in our study apoptosis induction in amelanotic melanoma cells may be also associated with the displace Bim from Mcl-1. Additionally, it was previously reported that MIM1 is able to increased levels of displaceable BH3-only proteins, such as Bim, in complex with overexpressed antiapoptotic Mcl-1 protein [17].

Reuland et al. [16] demonstrated that melanoma cells with lowered Mcl-1 expression are markedly susceptible to the BH3 mimetic - ABT-737 treatment. On the other hand, the melanoma cells with high level of Mcl-1 protein exhibited a resistance to ABT-737 treatment alone. This phenomenon may be explained by the fact that the tested BH3 mimetic inhibits only Bcl-2, Bl-xl and Bcl-w proteins and not Mcl-1 [16, 30]. Additionally, Wroblewski et al. [19] reported a significant decrease in the melanoma cell viability after exposure to obatoclax, a small molecule inhibitor which disables Bcl-2-, Bcl-xl-, Bcl-w-, and also Mcl-1. Taken together, it seems that the Mcl-1 protein inhibition is necessary and sufficient to exterminate melanoma cells. The selectively interaction with Mcl-1 protein is also more safe than inhibition of several antiapoptotic Bcl-2 members, because proteins, like Bcl-2 and Bcl-xl are responsible for the normal activity of melanocytes, fibroblasts and morphotic blood elements [31, 32].

To the best of our knowledge, there have been no previous studies investigating the effect of MIM1 on human melanoma cells. The proapoptotic activity of MIM1 was examined on leukemia cells. Cohen et al. [17] showed that MIM1 is able to selectively inhibit Mcl-1 and induce cell death in an Mcl-1-dependent leukemia cell line. Moreover, the authors observed synergistic cytotoxic effect when MIM1 was co-applied with another BH3 mimetic ABT-737. Whereas, another study presented that in case of non-small cell lung cancer which exhibit high expression of Mcl-1protein the ability of MIM1 to induce cell death is limited [33]. The observed susceptibility of different cancer cells for MIM1 treatment indicates that proapoptotic effect of this small molecule may depend not only on the Mcl-1 expression but also on specification of cell type.

Effectiveness of the anticancer therapy depends on their specific cytotoxicity, with damaging only cancer cells and exhibit low general toxic effects. Therefore, the resistance of non-malignant fibroblasts to MIM1 treatment [17] suggests that it could be a safe apoptosis-inducing compound.

The data presented herein clearly provide the first evidence that the use of Mcl-1 inhibitor – MIM1 contributes to the loss in the amelanotic melanoma cells viability, GSH depletion and DNA fragmentation which indicates that the tested Mcl-1 inhibitor exhibits high antitumor activity towards melanoma cells. Moreover, MIM1 intensified proapoptotic properties of DTIC and the combination of both agents induced cell death much earlier than MIM1 or DTIC alone. Summarizing, presented data indicate that blocking of Mcl-1 protein may be a potential important target in malignant melanoma treatment, and may constitute the basis for further in vitro studies with the use of MIM1 as the new anti-melanoma agent.
